# Factors Associated with Successful Response to Balloon Decompressive Adhesiolysis Neuroplasty in Patients with Chronic Lumbar Foraminal Stenosis

**DOI:** 10.3390/jcm8111766

**Published:** 2019-10-23

**Authors:** Yul Oh, Doo-Hwan Kim, Jun-Young Park, Gyu Yeul Ji, Dong Ah Shin, Sang Won Lee, Jin Kyu Park, Jin-Woo Shin, Seong-Soo Choi

**Affiliations:** 1 Department of Anesthesiology and Pain Medicine, Asan Medical Center, University of Ulsan College of Medicine, Seoul 05505, Korea; dhdbf@hanmail.net (Y.O.); knaaddict@gmail.com (D.-H.K.); anesthesia.pains@gmail.com (J.-Y.P.); sjinwoo@hotmail.com (J.-W.S.); 2 Department of Neurosurgery, Spine and Joint Research Institute, Guro Cham Teun Teun Hospital, Seoul 08392, Korea; jivago91@daum.net; 3 Department of Neurosurgery, Spine and Spinal Cord Research Institute, Severance Hospital, Yonsei University College of Medicine, Seoul 03722, Korea; shindongah@me.com; 4 Department of Neurosurgery, Yonsei Barun Hospital, Seoul 07013, Korea; goodi06@hanmail.net; 5 Department of Neurosurgery, Himchan Hospital, Bupyeong 21399, Korea; foryou94@hanmail.net

**Keywords:** lumbar foraminal stenosis, lumbar radicular pain, balloon catheter, epidural adhesiolysis, neuroplasty

## Abstract

Epidural neuroplasty, often called percutaneous epidural adhesiolysis, is often performed in refractory patients with chronic lumbar radiculopathy or neurogenic claudication. Recent studies have showed that decompressive adhesiolysis with an inflatable balloon catheter (balloon neuroplasty) is efficient in patients who experience refractory pain from epidural steroid injection or even epidural neuroplasty with a balloon-less catheter. However, exact indications or predictive factors for epidural balloon neuroplasty have not been fully evaluated. Therefore, to assess associated factors that could affect a favorable outcome, we analyzed a prospectively collected multicenter cohort of patients with chronic refractory lumbar foraminal stenosis after balloon neuroplasty. At the 6-month point in follow-up, 92 (44.4%) patients among 207 subjects were classified as successful responders according to a robust combination of outcome measures. Multivariate logistic regression analysis also showed that mild grade lumbar foraminal stenosis may be an independent factor associated with a successful response 6 months after balloon neuroplasty (odds ratio = 2.829; 95% confidence interval = 1.351–5.923; *p* = 0.006). However, we found that there were 29.4% and 24.6% successful responders with moderate and severe foraminal stenosis, respectively. Attempting balloon neuroplasty in refractory lumbar foraminal stenosis, especially mild grade, may be worthwhile.

## 1. Introduction

Epidural steroid injections are suggested to provide short-term symptom relief in patients with lumbar radiculopathy or neurogenic claudication, but benefits are not seen in all patients or are still inconsistent [1,2]. However, in these refractory cases, epidural neuroplasty, often called percutaneous epidural adhesiolysis, is currently performed using a shear-resistant catheter (the Racz-type catheter) or with a more steerable navigation catheter [3,4]. The effectiveness of percutaneous epidural neuroplasty is relatively well-established in these chronic refractory cases [5,6]. There are also previously investigated factors like hyaluronidase administrated, large volume injection and using hypertonic saline to favor a successful procedure outcome [7]. In addition, previous studies have showed that decompressive adhesiolysis using an inflatable balloon catheter (balloon neuroplasty) is efficient in patients who experience refractory pain from epidural steroid injection or even epidural neuroplasty with a balloon-less catheter [8,9]. Although previous studies have showed that spondylolisthesis, previous lumbar spinal surgery, and foraminal stenosis may be associated with outcomes after percutaneous epidural adhesiolysis with a balloon-less catheter [3], preprocedural patient factors associated with positive outcomes after percutaneous neuroplasty have not been clearly investigated. Recently, Kim et al. found that lumbar foraminal stenosis caused primarily by degenerative disc herniation may be an independent factor for favorable outcome after transforaminal balloon neuroplasty [9]. Choi et al. found that co-existing lower back pain and possible neuropathic conditions such as diabetes mellitus may be independent factors of poor outcome from decompressive adhesiolysis and neuroplasty with an inflatable balloon catheter in patients with lumbar radicular pain [10].

However, exact indications or predictive factors for epidural balloon neuroplasty have not been fully evaluated. Therefore, we aimed to assess associated factors and preprocedural findings that could affect the outcome of decompressive adhesiolysis and neuroplasty with an inflatable balloon catheter procedure in spinal stenosis patients with chronic refractory pain.

## 2. Materials and Methods

We analyzed a prospectively collected multicenter cohort to evaluate associated factors with successful outcome after epidural balloon neuroplasty [11]. Data was collected from the clinics of five different hospitals in the Republic of Korea (three spine-specialty hospitals and two university-affiliated teaching hospitals). This study was conducted in accordance with the Declaration of Helsinki. All participants gave informed consent and the study protocol was reviewed by the ethics committees or investigational review boards (approval number 2016-0228) at each participating site. This study was registered in the Clinical Research Information Service in Republic of Korea (KCT 0002280).

### 2.1. Participants

Between July 2015 and April 2018, a total of 317 patients with chronic lumbar foraminal spinal stenosis, aged 20 years or older, suffering from intractable lumbar radicular leg pain and/or lower back pain for more than 3 months were examined to ascertain their eligibility. A comprehensive assessment of medical history and a physical examination were done on every patient to exclude confounding disease as another cause of pain. To ascertain the diagnosis of spinal stenosis and determine the grade or level of spinal stenosis, lumbar magnetic resonance imaging (MRI) was performed on all patients. The degree of lumbar foraminal stenosis was analyzed based on MRI findings, as described in a previous study [12]. In brief, MRI reporting was graded using categories of the exiting nerve root compression. Grade 0 refers to normal neuroforamen, Grade 1 for mild foraminal stenosis showing perineural fat obliteration in two opposing directions, Grade 2 for moderate foraminal stenosis showing perineural fat obliteration in four directions, and Grade 3 for severe foraminal stenosis showing morphologic changes in the nerve root [12].

We analyzed data from patients who underwent the ballooning decompressive adhesiolysis procedure in the stenotic foramina and retrodiscal area of the lumbar spine by the methods described below.

The inclusion criteria were as follows: patients who had been diagnosed with foraminal stenosis from lumbar MRI with a related chronic (more than 3 months) lumbar radicular pain, with or without lower back pain; those who were refractory to conservative treatment; and those who had failure of interlaminar epidural steroid injection or transforaminal epidural block (less than 50% pain improvement or lasting less than one month) are enrolled in this study.

The exclusion criteria were as follows: patient refusal to participate in this study, age less than 20 years old, axial pain such as in lumbar facet syndrome or myofascial pain syndrome, previous steroid injection within 12 weeks prior, progressive neurological deficits or motor weakness, uncontrollable or unstable opioid use, previous side effects to steroids, coagulopathy, signs of infection, pregnancy or nursing, allergy to local anesthetics or contrast dye solutions, history of previous spine operation, or an unstable medical or psychiatric condition.

### 2.2. Procedure: Percutaneous Epidural Neuroplasty Using an Inflatable Balloon Catheter

**Figure 1:**
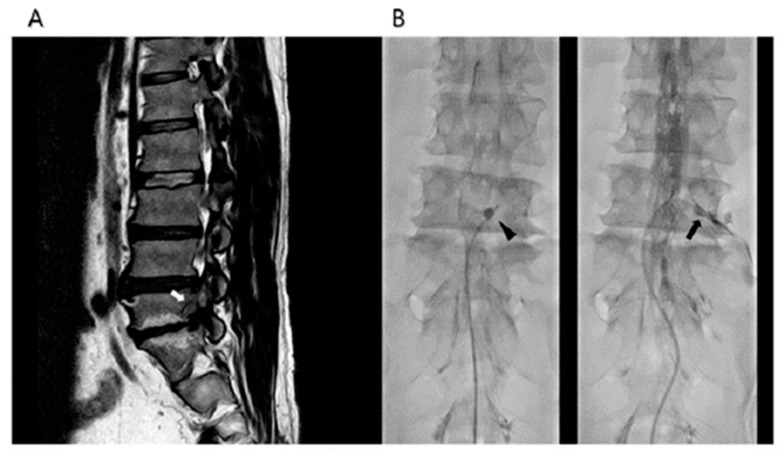
Percutaneous epidural adhesiolysis combined with balloon decompression in a patient with right L5-S1 foraminal stenosis. (**A**) A lumbar magnetic resonance image of a 59-years- old man with right lower extremity neurogenic claudication shows a moderate grade of stenosis in the right L5-S1 lumbar foramen (white arrow). (**B**) Anteroposterior fluoroscopic view showing the inflatable balloon neuroplasty catheter placed in the right L5-S1 intervertebral foramen and the balloon filled with the contrast medium (black arrow head). Foraminal stenosis is visualized by the degree of balloon distortion (black arrow).

After sterile preparation before the procedure, a local anesthetic (Lidocaine, 2%) was injected under the skin and soft tissue. A 10-gauge guide needle, specially designed for preventing various types of potential damage by a catheter during catheter manipulation, was advanced through the sacral hiatus. The guide needle was gently introduced via the sacral hiatus under fluoroscopic image guidance. Consequently, about 8 mL of diluted contrast medium (Omnipaque, Nycomed Imaging AS, Oslo, Norway) was injected using the guide needle. The diluted contrast medium was prepared by mixing 4 mL of pure contrast medium and 4 mL of 1% lidocaine. If intravascular injection was detected, the needle was repositioned. After suitable identification via an epidurogram of the target areas, a catheter (ZiNeu^®^, JUVENUI, Seoul, Korea) was advanced via the guide needle to the filling defects or pathologic featured area determined based on MRI findings and comprehensive assessment of symptoms before the procedure. The planned sites were confirmed to be symptom-related filling defects by epidurogram and were targeted by mechanical adhesiolysis and balloon decompression via catheterization using the ZiNeu catheter. The epidural adhesiolysis and balloon decompression were performed via side-to-side positioning of the catheter with intermittent balloon inflating (Figure 1). The balloon of the catheter was prepared by filling 0.13 mL of contrast agent with a 1 mL Luer-Lock syringe (BD Medical, Franklin Lakes, NJ, USA), and ballooning was limited to 5 s each time [11,13]. For safety reasons, the balloon inflation time was adjusted based on the degree of pain caused by the procedure: if the patient complained of severe pain during balloon inflation, no further decompression was attempted. The catheter moved only when the balloon deflated.

All procedures were performed by experienced physicians. The patients were followed up in the outpatient department of the clinic at 1, 3 and 6 months after the procedure.

### 2.3. Outcome Assessments

Basic characteristics such as age, gender, body mass index, duration of pain, pain intensity in leg and back, grade of foraminal stenosis, Oswestry disability index (ODI), and medication quantification scale (MQS) were obtained for analysis. The outcome evaluation was performed 6 months after the procedure. All participants were instructed and evaluated using the following scales: an 11-point numerical rating scale from 0 (no pain) to 10 (worst possible pain) to determine the intensity of both leg and lower back pain; the Korean version of the 10-item ODI questionnaire (range, 0–100; 0 = no disability) to determine physical functional status; and the Beck depression inventory to assess emotional status. The MQS III was also measured to assess changes in the usage of medication [14]. The global perceived effect (GPE) after the procedure according to the 7-point Likert scale was also measured to analyze the patient’s satisfaction and improvement after balloon adhesiolysis [15]. In addition, possible complications associated with the procedure were recorded, and all side effects were further evaluated at follow-up visits.

### 2.4. Definition of Successful Responses 

We define a responder group according to previous studies [10,11], with some modifications, as: (1) 50% (or ≥ 4 point) decrease of NRS from baseline, no increase from baseline ODI and MQS, and ≥ 4 points on the GPE scale; or (2) ≥ 30% (or ≥ 2-point) decrease of NRS from baseline together with any one of the following criteria: ≥ 30% (or ≥ 10 point) decrease in ODI from baseline, ≥ 5 points on the GPE scale, or ≥ 25% reduction from the baseline MQS. According to this definition of response, patients were divided into responder and non-responder 6 months after the balloon neuroplasty.

### 2.5. Statistical Analysis

Continuous demographic data from the non-responders and successful responders were compared by using the Student’s *t*-test or the Mann–Whitney *U*-test and were documented as means with standard deviations or medians with interquartile ranges as appropriate. Categorical demographic data were compared using a chi-square test or a Fisher’s exact test. By using univariate and multivariate regression, the factors associated with a successful response 6 months after adhesiolysis with an inflatable balloon catheter were analyzed. The most relevant factors associated with successful responses were included in the univariate logistic regression analysis. The inclusion of variables in the final multivariate logistic regression analysis to evaluate independent factors associated with successful responses was based on biological plausibility, clinical importance, and statistical considerations. The quality of fit of the model was assessed with the Hosmer-Lemeshow test. A two-tailed *p*-value < 0.05 was considered to indicate a statistically significant difference. The data were analyzed by using the Statistical Package for the Social Sciences Version 21.0 (SPSS Inc., Chicago, IL, USA).

## 3. Results

**Figure 2:**
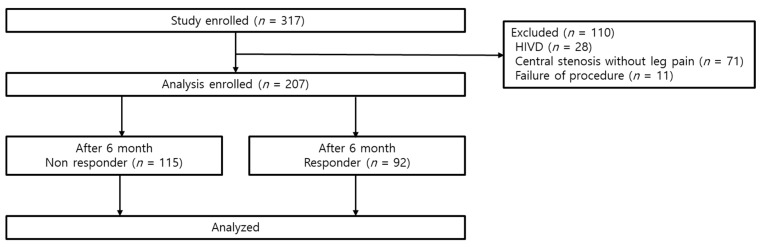
Study flow diagram.

A total of 317 patients were enrolled in this study. One hundred and ten patients were excluded due to herniated lumbar intervertebral discs (HIVD; *n* = 28), central stenosis without radiating pain (*n* = 71), and procedure failure (*n* = 11). Finally, 207 subjects were enrolled in analysis. Six months into follow-up, 115 (55.6%) patients were classified into the non-responder group and 92 (44.4%) patients were classified into the responder group according to the definition described above (Figure 2).

The demographic characteristics of non-responders and responders are summarized in Table 1. There was a significant difference in the degree of lumbar foraminal stenosis grade between the two groups. On average, the non-responder group contained more moderate and severe lumbar foraminal stenosis subjects. Otherwise, no significant differences in other baseline characteristics were observed between the two groups. In univariate logistic regression analysis, we presumed a meaningful statistical *p*-value below 0.1 (*p* < 0.1). We found three statistically meaningful factors: age (*p* = 0.078), mild foraminal stenosis grade (*p* = 0.003), and ODI (*p* = 0.003). After adjusting demographic differences for multivariate regression analysis, the association between mild grade foraminal stenosis and the responder group showed a statistically significant (*p* = 0.006) association (Table 2). The association between age and ODI with a successful response was no longer significant. A mild grade lumbar foraminal stenosis is an independent factor associated with successful responses 6 months after decompressive adhesiolysis with an inflatable balloon catheter (odds ratio = 2.829; 95% confidence interval = 1.351—5.923; *p* = 0.006) (Table 2). Cumulative lists and rates of the observed complications during the decompression and adhesiolysis with an inflatable balloon catheter are shown in Table 3. However, none of the patients experiencing complications had persistent neurologic abnormalities, and all were discharged after bed rest for a short duration.

## 4. Discussion

Several studies have reported on the effectiveness and clinical outcomes of percutaneous decompressive adhesiolysis with an inflatable balloon catheter in patients with refractory lumbar spinal stenosis [8,9,10,11,16]. It can now be considered a feasible option in a patient who has had inadequate symptom relief despite conventional treatment with epidural steroid injection or percutaneous epidural adhesiolysis with a balloon-less catheter. Clearly, identifying the factors associated with a successful outcome of the interventional procedure is important in selecting a treatment option for symptom relief, improvement of quality of life, and patient satisfaction.

Previously, various studies have reported certain factors associated with the effectiveness of percutaneous epidural adhesiolysis. Patients with conditions including spondylolisthesis, previous lumbar spinal surgery, and foraminal stenosis may be expected to have a lower probability in reaching satisfactory outcomes with percutaneous epidural neuroplasty with a balloon-less catheter [3,17]. In percutaneous balloon neuroplasty for the management of chronic lumbar foraminal stenosis, a foraminal stenosis owing to a degenerative herniated intervertebral disc had reported favorable associated factors after the balloon procedure [9]. In addition, diabetes and combined lower back pain with lumbar radicular pain may be negatively associated factors with percutaneous balloon neuroplasty [10]. On the other hand, several previous reports did not find an association of foraminal stenosis grade with clinical outcome after balloon neuroplasty [9,10,11]. In the present study, multivariate logistic regression analysis revealed mild grade foraminal stenosis in lumbar MRI to be an independent factor positively associated with successful responses to decompressive adhesiolysis by using inflatable balloon catheter, which had not been revealed in previous studies. This discrepancy may be regarded as a limitation of a small-sample-size study [18] or a study of patients with mixed diagnoses (blended etiology of central type stenosis and foraminal type stenosis). A moderate or severe grade of lumbar foraminal stenosis suggests a more progressed state than mild stenosis and may be deemed more difficult to manage. In that sense, the results of this study may be taken for granted. However, it did not seem that mild lumbar foraminal stenosis is an exclusive indication. Actually, in the present study, we found that there were 29.4% and 24.6% successful responder patients in moderate and even in severe foraminal stenosis, respectively. This indicates that attempting decompressive adhesiolysis with a balloon catheter in symptomatic moderate and severe foraminal stenosis patients may be worthwhile. The grade of lumbar foraminal spinal stenosis may be a good predictive factor for successful response instead of an indicative determinant of whether to attempt the inflatable balloon catheter neuroplasty or not. The present results suggest that decompressive adhesiolysis with an inflatable balloon catheter can be effective in refractory pain after a conventional epidural block or even neuroplasty with a balloon-less catheter.

This study has some limitations. Firstly, we did not evaluate neurogenic claudication, which is a representative symptom of lumbar foraminal spinal stenosis [19]. It has been reported that severity of claudication may be a risk factor of poor outcome in conservative treatment of spinal stenosis [20]. However, in the present study, we assessed ODI for functional and physical status, although some authors insisted that the value of ODI may be insufficient for a precise evaluation of claudication grade and of measuring outcome improvement. Further study is required with a delicate tool to represent the severity of neurogenic claudication [21,22]. Secondly, there remains a vague correlation with the degree of symptoms and stenosis grade in radiologic finding [10,23]. Neither severe nor mild lumbar foraminal stenosis in MRI findings meaningfully relates to the degree of clinical symptoms. In the light of these clinical considerations, a well-designed symptom-related outcome analysis study of percutaneous decompressive adhesiolysis using an inflatable balloon catheter in lumbar spinal stenosis is also recommended in the near future.

## 5. Conclusions

Attempting percutaneous decompressive adhesiolysis using an inflatable balloon catheter in refractory lumbar foraminal stenosis regardless of severity may be worthwhile. Particularly, mild grade lumbar foraminal stenosis seen in MRI may be a favorable independent factor associated with a successful outcome after percutaneous balloon neuroplasty. 

## Figures and Tables

**Table 1:** Patient characteristics.

	Non-Responder (*n* = 115)	Responder (*n* = 92)	*p* Value
Age (years)	64.1 ± 12.5	60.9 ± 13.1	0.077
Sex (male/female)	64/51 (55.6/44.3)	48/44 (52.1/47.9)	0.618
Height (cm)	161.3 ± 17.8	161.2 ± 19.3	0.943
Weight (kg)	65.3 ± 12.1	65.1 ± 9.3	0.887
Body mass index (kg/m^2^)	24.1 ± 4.3	24.2 ± 3.7	0.843
Pain duration (months)	12.0 (6.0–24.0)	12.0 (8.5–24.0)	0.468
Foraminal stenosis grade, *n* (%)			0.005
Mild	36 (31.3)	49 (53.3)	
Moderate	44 (38.3)	27 (29.3)	
Severe	35 (30.4)	16 (24.6)	
Baseline pain intensity (NRS)			
Back	5.7 ± 2.3	6.1 ± 1.9	0.149
Leg	6.7 ± 1.8	6.5 ± 1.8	0.426
Oswestry Disability Index	31.8 ± 11.4	29.1 ± 9.9	0.081
Medication quantification scale	5.9 ± 5.9	7.0 ± 6.0	0.402

Data are expressed as mean ± standard deviation (SD), medians (interquartile range), or numbers (%). NRS = numeric rating scale.

**Table 2:** Logistics regression analysis of factors associated with successful response at 6 months after decompressive adhesiolysis neuroplasty using an inflatable balloon catheter.

	Univariate			Multivariate		
	OR	95% CI	*p* Value	OR	95% CI	*p* Value
Age	0.981	0.960–1.002	0.078	0.994	0.970–1.019	0.642
Sex						
Male	1 (*Ref*)					
Female	1.150	0.664–1.994	0.618			
BMI	1.007	0.940–1.079	0.842			
Pain Duration	1.003	0.995–1.011	0.492			
Foraminal Stenosis Grade						
Severe	1 (*Ref*)			1 (*Ref*)		
Moderate	1.342	0.627–2.874	0.448	1.289	0.592–2.808	0.523
Mild	2.977	1.433–6.187	0.003	2.829	1.351–5.923	0.006
Pain Intensity (NRS)						
Back	1.100	0.966–1.251	0.149			
Leg	0.939	0.806–1.095	0.424			
Oswestry Disability Index	0.976	0.950–1.003	0.083	0.980	0.952–1.009	0.181
MQS	1.033	0.958–1.114	0.398			

CI = confidence interval; MQS = medication quantification scale; NRS = numeric rating scale; OR = odds ratio; Ref = reference.

**Table 3:** Cumulative list and rate of the observed complications during the decompression and adhesiolysis with an inflatable balloon catheter.

Complication	Number (%)
Suspected Dura Puncture	8 (3.9)
Subdural Injection	4 (1.9)
Vascular Injection	3 (1.4)
Disc Injection	4 (1.9)
Hypotension	4 (1.9)
